# Dog Owners’ Perceptions of Canine Body Composition and Effect of Standardized Education for Dog Owners on Body Condition Assessment of Their Own Dogs

**DOI:** 10.3390/vetsci10070447

**Published:** 2023-07-08

**Authors:** Sanna Gille, Hanna Fischer, Sanna Lindåse, Linda Palmqvist, Julia Lärka, Sara Wolf, Johanna Penell, Josefin Söder

**Affiliations:** Department of Clinical Sciences, Faculty of Veterinary Medicine and Animal Science, Swedish University of Agricultural Sciences, P.O. Box 7054, 75007 Uppsala, Sweden

**Keywords:** body composition, body condition score, BCS, dog, obesity, overweight, perception

## Abstract

**Simple Summary:**

Overweight is a growing problem in dogs worldwide and negative health effects associated with excess body weight are common. The body condition score (BCS) scale is a time- and resource-effective method to assess if a dog is overweight, but its precision among dog owners has been found to vary. The aim of this study was to investigate dog owners’ perceptions of various body compositions in dogs and evaluate if a short education on how to use the 9-point BCS scale might change these perceptions. This study included one survey and one clinical study of Swedish dog owners. In the indirect assessment based on photos, normal-weight dogs were underestimated by three-quarters of dog owners, and about half of the dog owners underestimated overweight dogs. Before receiving the standardized education, one-third of the owners underestimated the body composition of their own dogs, mainly for dogs with excess adiposity. The dog owners responded well to the practical education given and, thereafter, performed assessments comparable to veterinary health care personnel. These results indicate that perception of what an “ideal weight” dog should look like is sliding and that the ability to identify overweight dogs might be limited when owners evaluate body composition without previous education.

**Abstract:**

Overweight in dogs is an increasing problem, with a prevalence of about 30% in Sweden. To prevent the negative health effects of overweight, it is important to identify and treat canine overweight. Dog owners are essential for such interventions. The aim of this study was to evaluate dog owners’ perceptions of various canine body compositions via indirect assessment based on photos and direct assessment of their own dogs. A second aim was to evaluate the effect of a standardized practical education for dog owners on body condition score (BCS) assessment of their own dogs. The 9-point BCS scale was used, and two study samples were recruited: one was a survey sample where 564 dog owners assessed the BCS of dogs using photos, and one sample was a separate clinical sample where 82 dogs were assessed by their owners and by veterinary health care personnel. The initial BCS assessment by the dog owners in the clinical sample (mean ± SD) was significantly lower (4.6 ± 1.0) than the BCS assessed by the veterinary health care personnel (5.2 ± 1.1), but the owners improved significantly after receiving the standardized education (5.1 ± 1.0) (both *p* < 0.0001) and performed as accurately as the veterinary health care personnel (*p* = 0.99). The results should be verified in the broader dog owner population based on a randomized selection of participants. “Weight blindness”, defined here as an underassessment of normal-weight dogs and an inability to identify overweight dogs, is likely to have a negative impact on canine overweight prevalence. Deeper knowledge about dog owners’ perceptions can inform the development of new strategies to help prevent and manage canine overweight, whereof standardized practical education on BCS assessment is shown here to be one example.

## 1. Introduction

Canine obesity is highly prevalent and an increasing problem in various countries [1,2,3,4,5,6]. In the Swedish dog population, the overweight prevalence is approximately 30% [2,7]. Overweight dogs may experience a decreased quality of life and a shortened lifespan [8,9,10,11,12]. In addition, overweight dogs have a higher risk of developing comorbidities, such as metabolic dysregulations and joint diseases with lameness [9,13,14], but such comorbidities may be reversed if overweight is reduced [15,16].

Body weight alone is not sufficient to conclude if a dog has an ideal body condition or is under- or overweight as body weight is constituted of both lean and fat mass [17,18], and must be related to the overall size of the dog. Instead, assessment of body condition score (BCS) using palpation in combination with visual inspection is a necessity to accurately determine body condition status in dogs [18]. The BCS system is a validated semi-quantitative method which categorizes dogs on a 9-point scale from very thin to very obese, and has many advantages, both clinically and in research, such as being quick to administer, not requiring any equipment, and having high reproducibility [17,18,19,20]. The BCS system is well known and used by many veterinary health care personnel as it gives important information about an animal’s energy status. However, within the dog owner community, the precision of BCS assessment has been reported to vary, with an underassessment of body condition, i.e., categorize an overweight dog as normal weight, as the main risk [4,21,22,23].

The definition of canine obesity as a disease is a relatively new concept. In 2019, the Global Pet Obesity Statement proposed a uniform classification of canine obesity and emphasized the importance of spreading education on how to use the 9-point BCS scale to accurately assess body condition [18]. It has been shown that obesity in humans follows social patterns; the more prevalent overweight is among people we socialize with, the more likely we unconsciously perceive overweight as being normal [24]. The human ideology of advocating a positive body image suggests that you should embrace your body despite size or shape [25], which has been shown crucial for sustained health-related human behaviors [26]. Pet dogs, however, are dependent on their owners’ awareness of their potential overweight to make appropriate adjustments in energy intake and/or physical activity level to retain or remain at an ideal body composition. The high prevalence of overweight dogs today may shift the experience of normality. If the perception of what an ideal canine body condition looks like is sliding toward an underestimation of normal-weight dogs’ body composition, there is a subsequent risk for further increase in overweight prevalence in dogs in general.

Dog owners are an important and innate target group for educational efforts not only because they are the everyday caretakers of dogs but also because of the shared lifestyle with their pets [2,27,28,29,30,31]. Dog owners’ body composition and/or attitudes toward healthy diets and physical activity are factors shown to influence BCS in dogs [2,31]. Owners are central to any preventive actions or successful weight loss programs in their dogs; but if they are unaware of the overweight problem, they will not be able to engage [32,33]. Awareness of canine overweight and how to assess it are, therefore, important factors to address in dog owner populations. The aim of this study was to evaluate dog owners’ perceptions of various canine body compositions via indirect assessment based on photos and direct assessment of their own dogs. A second aim was to evaluate the effect of a standardized practical education for dog owners on body condition score (BCS) assessment of their own dogs.

## 2. Materials and Methods

### 2.1. General Study Design

This study included two different study samples of Swedish dog owners: one sample where data were collected through an online survey (“survey population”), and one sample where dog owners and their respective dog/dogs participated in an experimental study (“clinical population”). All human participants in the survey, as in the clinical population, had to be 18 years or older. Both study samples were non-probability samples (i.e., convenience samples) based on voluntary participation.

#### 2.1.1. Survey Sample: Indirect Assessment of Dogs Using Photos

An online survey was distributed to Swedish dog owners and was available online through social media for seven days. It contained questions on the responders’ knowledge about the BCS system (see Table S1: Survey questions and research raw data) and investigated owner perceptions of different canine body compositions via indirect assessment based on photos whereby the respondents were asked to assess the BCS of four dogs by choosing a score (1–9) (see Figure S1: Dog photos). In addition to the dog photos, the respondents were given a short description of different BCSs (1–9) in Swedish [17], but no schematic BCS illustration was provided in order to record “perceptions” of canine body composition, i.e., without guiding pictures from a scale or previous education. In the Swedish description of the scores given, BCSs of 1–3 were grouped and described as underweight, BCSs of 4–5 were grouped and described as normal weight, a BCS of 6 was described as slightly overweight, a BCS of 7 was described as overweight, and BCSs of 8–9 were grouped and described as obese. All questions in the survey were compulsory, and only fully completed surveys were included in the descriptive statistics and statistical analyses.

All four dogs shown in the photos in the survey were male Labrador Retriever dogs of the type for shows, which were previously assessed in vivo by the primary investigator (J.S.) The dogs had a BCS of 5–8 (normal weight to obesity) and were photographed from above in a standardized setting (in the same room and position and by the same photographer).

#### 2.1.2. Clinical Sample: Direct Assessment of Own Dogs Based on Predefined Oral Descriptions Followed by BCS Assessment after Standardized Education

A clinical study that investigated the accuracy of in vivo body condition assessment by dog owners was completed with Swedish dog owners and their dogs, who were recruited through social media from three regions located in southern, middle, and northern Sweden (Höör, Uppsala, and Umeå). The participating dog owners participated with one or several dogs. The dogs were allowed to be of any breed, sex, age, neuter status, and health status, but they were obligated to show no signs of aggression whilst being handled by strangers. The clinical study was performed by two veterinary nursing students in their last semester of their Bachelor Veterinary Nursing Program at the Swedish University of Agricultural Sciences, Uppsala. The students had received previous education on BCS assessment and specific BCS calibration with an experienced BCS assessor (J.S.) before data collection. All individual dogs were assessed for BCS three times: two times by their respective owner (before and after the standardized education) and one time by of one of the two students. The students will hereafter be referred to as “veterinary health care personnel” in this publication.

The clinical sample’s data collection started with investigating the owners’ perceptions through a direct assessment of their own dogs based on predefined oral descriptions, where the dog owners described the body composition of their dogs as “underweight”, “slightly underweight”, “normal weight”, “slightly overweight”, “overweight”, or “obese”. Thereafter, the dog owners received a standardized BCS education where they were shown the 9-point BCS scale from World Small Animal Veterinary Association [34] in Swedish, and a short standardized oral description of the BCS scale was given (5–10 min). The palpable anatomical localizations of the ribs, waist, and abdominal line were demonstrated on the dogs and, thereafter, the owners performed their BCS assessment of their dog/dogs without any further guidance. The dog owners were given a laminated instruction sheet about the BCS scale and, on the backside of the sheet, the standardized information they had received orally was printed (see Supplementary Material S1: Standardized information). Lastly, the veterinary health care personnel made their BCS assessment of the dog/dogs without knowing which BCS each owner had picked. The dog owners participating with more than one dog were asked to describe all their dogs using the predefined oral descriptions before receiving the BCS education; thereafter, they assessed all their dogs before a BCS assessment was made by the veterinary health care personnel (see Table S1: Survey questions and research raw data).

### 2.2. Data Processing

The data related to dog owners (survey population) or to dog owners and their dogs (clinical population) were analyzed descriptively as proportions, percentages, and/or by using statistical models.

For the survey sample, the expert who had previously performed the in vivo BCS assessment of the dogs in the photos was considered “the primary investigator”, and the expert’s scores were compared with the indirect assessment based on photos performed by the dog owners (the survey population did not receive any education). Free-text answers in the survey regarding negative consequences for overweight dogs stated by the dog owners were grouped as “the locomotor apparatus”, “heart- and coronary disease”, “insulin resistance/diabetes mellitus”, “organ related disease”, “less physically active”, “increased risk for diseases or injuries”, “shortened lifespan”, “reduced quality of life”, and “other negative effects”.

For the clinical sample, the veterinary health care personnel were considered to be the primary investigators, and their scores were compared with the direct assessment performed by the owners on their own dogs based on predefined oral descriptions and the owners’ BCS assessment after the standardized education. The predefined oral descriptions were translated into BCS scores for data processing to enable statistical analyses. The translation was made as follows: “underweight” (BCS = 1.5), “slightly underweight” (BCS = 3), “normal weight” (BCS = 4.5), “slightly overweight” (BCS = 6), “overweight” (BCS = 7), and “obese” (BCS = 8.5) [17]. The data from each owner for each dog they included were considered one observation.

### 2.3. Statistical Analyses

For statistical analyses, the software GraphPad Prism (GraphPad Prism 5.0, San Diego, CA, USA) and SAS (SAS 9.4 Institute Inc., Cary, NC, USA) were used. All data were normally distributed based on an evaluation of the appearance of residuals in SAS or based on D’Agostino and Pearson omnibus normality test in GraphPad Prism. The level of significance for all statistical analyses was set to *p* < 0.05, and the results are presented as mean ± SD or mean ± SEM.

#### 2.3.1. Survey Sample

For the indirect assessment based on photos, a Chi-square test for trend was used to analyze differences in proportions (answers were grouped as “equivalent”, “under-assessment”, and “over-assessment”) among all survey participants and all four dogs shown in the photos (grouped as BCSs of “5”, “6”, “7”, and “8”). In this analysis, the exact score set by the primary investigator was considered the “equivalent assessment”.

A Chi-square test for trend was also used to analyze differences in proportions (answers were grouped as “equivalent” and “under/over-assessment”) between owner age groups (grouped as “18–25 years”, “26–40 years”, “41–60 years”, and ≥61 years”) regarding the normal-weight dog (BCS = 5) and the obese dog (BCS = 8). A Chi-square test for trend was also used to analyze differences in proportions (answers were grouped as “equivalent” and “under/over-assessment”) between groups of owners with varying knowledge of the BCS scale (answers were grouped as “Yes” or “No” based on familiarity) regarding the normal-weight dog (BCS = 5) and the obese dog (BCS = 8). In the analyses including the factors “owner age” and “previous knowledge”, BCSs of 4–5 and BCSs of 8–9, defined based on the scoring scale of the BCS for normal-weight and obese dogs [17], were considered the “equivalent assessments”.

#### 2.3.2. Clinical Sample

The body condition assessments collected from the clinical sample were evaluated using a mixed model random analysis in SAS [35], where both “dog” and “owner” were set as the random effects. The purpose of the mixed model was to compare the BCS assessments performed on the same dog (in triplicate), with the scores as the dependent variable. The three assessments (time points) included in the mixed model were the three body condition assessments of each dog: (1) owner perception in the “direct assessment based on predefined oral descriptions”, (2) owner “BCS assessment after education”, and (3) “BCS assessment performed by veterinary health care personnel”. The explanatory factor tested was “owner age” (grouped as “19–37 years”, “38–55 years”, and “56–73 years”), as this factor previously had been shown to affect canine body composition [30] and, therefore, hypothetically could affect owner BCS assessments. The factor “previous dog ownership” (grouped as “no previous experience”, “1–3 former dogs”, and “4–12 former dogs”) was selected as the second explanatory factor as it was hypothesized that owner age could be a marker for number of owned dogs; alternatively, dog ownership experience could be independent of owner age. In the mixed model, the explanatory factors were defined as the independent variables, and the model analyzed differences between groupings of factors (i.e., effect of owner age and effect of number of previously owned dogs on owner assessments), differences between time points (i.e., comparison of owner perception with assessment made by the veterinary health care personnel and effect of owner education), as well as the interactions between the factors and time points in the overall and pairwise comparisons within the mixed model. Separate models were created for the two explanatory factors. Corrections for multiple comparisons were performed using Tukey–Kramer adjustment within the models.

A Chi-square test for trend was used to analyze differences between the selected explanatory factors: “number of previously owned dogs” (grouped as “no previous experience”, “1–3 former dogs”, and “4–12 former dogs”) and “owner age” (grouped as “19–37 years”, “38–55 years”, and “56–73 years”). Linear regression analysis was used to analyze the correlation between owner age and number of previously owned dogs as continuous variables.

Linear regression analysis and Student’s *t*-tests were used to analyze the effects of dog intrinsic factors, such as age, gender, and neuter status, on BCSs. Linear regression analysis was used to analyze the association between dog age and BCS (the BCS assessment by veterinary health personnel was used). Student’s *t*-tests were used to compare the BCSs of male and female dogs as well as the BCSs of intact and neutered dogs.

## 3. Results

### 3.1. Survey Sample

#### 3.1.1. Descriptive Data

In total, 952 dog owners started the online survey and 564 dog owners completed the questionnaire. The respondents were predominantly women (96%), and the most represented age group was 41 to 60 years of age (45%). Upper secondary school (50%) was the most common educational level, and the largest professional categories were human health care (21%) and veterinary health care (12%). The respondents had previously owned (mean ± SD) 7.0 ± 6.7 dogs (range 1–30). Forty percent (224/564) answered “yes” to the question if they knew what the BCS is and what it is used for. Of those respondents with self-perceived knowledge of the BCS, 84% correctly described the term related to body fat content in an open-ended follow-up question. Nine percent described that the term was related merely to body weight, and 7% described that the term was related to overall health. The descriptive statistics of the survey sample are shown in Table 1. The importance of dog owners’ ability to correctly assess the BCS of their dogs was rated by the respondents as (mean ± SD) 4.6 ± 0.6 (range 1–5), where a score of 5 represented “extremely important”.

**Figure 1:**
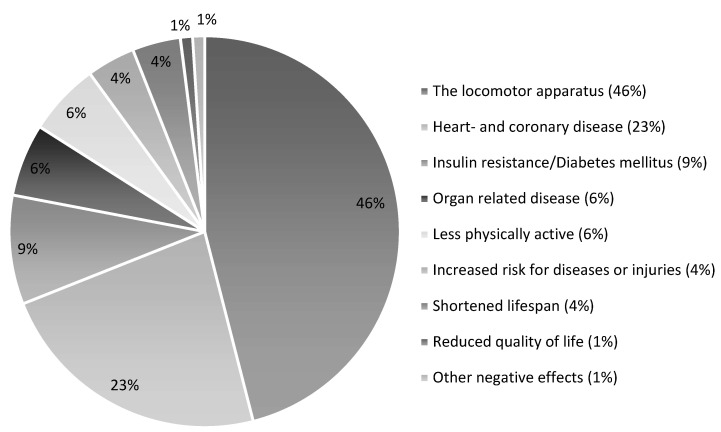
Pie chart presenting the proportions of negative consequences for overweight dogs stated by dog owners. The respondents were able to submit more than one answer to an open-ended question, and a total of 1061 answers were recorded from the 564 survey respondents. The recorded negative consequences were grouped into nine categories, as shown in the figure, and the proportions in percentage were calculated from the total sum of 1061 answers.

Almost all survey respondents (99%) agreed that being overweight may lead to negative consequences for dogs. In the open-ended follow-up question, the respondents were asked to give a few examples of negative consequences, and 1061 free-text answerers were recorded. The answers were grouped according to type of problem described, and the two largest groups were “joint problems” (46%) and “heart- and coronary diseases” (23%). The least frequent groups were “a shortened life span” (4%) and “a reduced quality of life” (1%) (Figure 1).

#### 3.1.2. Owner Perceptions: Indirect Assessment of Dogs in Photos

**Figure 2:**
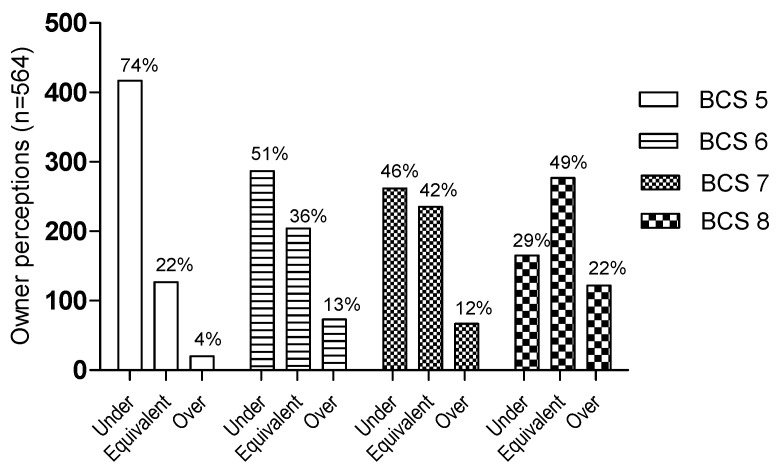
Descriptive statistics of owner perceptions in the indirect assessment based on photos. The results are divided into underassessment, equivalent assessment, and overassessment compared to the exact BCSs set by the primary investigator in the “in vivo” assessment of the same dogs, shown per body condition score (BCS = 5–8). Owner assessment of normal-weight dog (BCS = 5), slightly overweight dog (BCS = 6), overweight dog (BCS = 7), and obese dog (BCS = 8) are represented by different patterns of the bar charts, and data are shown in proportions (percentages) for each BCS category. The dog owners’ accuracy in the indirect assessment based on photos depends on the BCS of the assessed dog (Chi-square test for trend, *p* < 0.0001).

Of all survey respondents, 74% underestimated the BCS of the normal-weight dog (BCS = 5), and about half of the respondents could not identify a slightly overweight (BCS = 6) or an overweight dog (BCS = 7). On the contrary, as many as 71% could correctly identify an obese dog (BCS = 8) with an exact score or higher (Figure 2). Among all survey respondents, the dog with a normal weight (BCS = 5) was assessed as having a BCS of (mean ± SD) 3.9 ± 1.2. The dog with slight overweight (BCS = 6) was assessed as having a BCS of 5.5 ± 1.1, the overweight dog (BCS = 7) was assessed as having a BCS of 6.5 ± 1.0, and the obese dog (BCS = 8) was assessed as having a BCS of 7.8 ± 1.0. The dog owners’ accuracy in the indirect assessment based on photos depended on the BCS of the assessed dog (*p* < 0.0001), where the normal-weight dog was the dog that was most commonly underestimated, followed by the slightly overweight dog, while the obese dog was the dog that was most commonly accurately assessed, when compared to the exact in vivo assessment of the same dogs performed by the primary investigator (which was considered the gold standard method in the survey sample) (Figure 2).

For the survey respondents, both “age group” and “previous knowledge” of the BCS scale had an effect on the accuracy of owner perceptions in the indirect assessment based on photos. The respondents aged 61+ years showed a significantly lower proportion (51%) in terms of equivalent BCS assessment for the normal-weight dog (BCS = 5) compared to the other age groups (64–75%) (*p* = 0.0014). In the age group of 61+ years, the dog with a BCS of 5 was given a mean score of 3.5, and 46% assessed the normal-weight dog as being underweight (BCS = 1–3). For the obese dog (BCS = 8), the different age groups did not differ in proportion for equivalent assessment or underassessment (*p* = 0.15). The respondents with no previous knowledge of the BCS scale showed a significantly lower proportion (61%) in terms of equivalent BCS assessment for the normal-weight dog (BCS = 5) compared to the respondents with previous knowledge (77%) (*p* < 0.0001). For the obese dog (BCS = 8), previous knowledge of the BCS scale did not affect the proportion of equivalent assessment or underassessment (*p* = 0.15). Within all age groups, having “no previous knowledge” of the BCS scale was numerically more frequent than having “previous knowledge” (Table 2), but the proportions (Familiar: yes/no) did not differ significantly between different owner age groups (*p* = 0.09).

### 3.2. Clinical Sample

#### 3.2.1. Descriptive Data

In total, 64 dog owners participated in the study with 82 dogs, thus accounting for 82 observations. Thirteen dog owners participated in the clinical study with two dogs, one owner participated with three dogs, and one owner participated with four dogs. Of the dog owners participating with more than one dog, 7/13 (54%) showed systematic patterns in their assessment of their dogs, which were defined as constant under-assessment, equivalent assessment, or over-assessment.

The gender distribution is presented in Table 3. The age of the owners varied between 19 and 73 years, with a mean age of 47 years. When asked about their occupation, 32 stated that they were working, 13 were studying, 2 were unemployed, and 14 had retired. The dogs were of 41 different breeds (n = 58) or of mixed breeds (n = 24). No breed was overrepresented, with a maximum of four individuals within the same breed, and 46 dogs were females and 36 were males. Details of the clinical sample are shown in Table 3.

#### 3.2.2. Owner Perceptions: Direct Assessment of Own Dogs Based on Predefined Oral Descriptions

**Figure 3:**
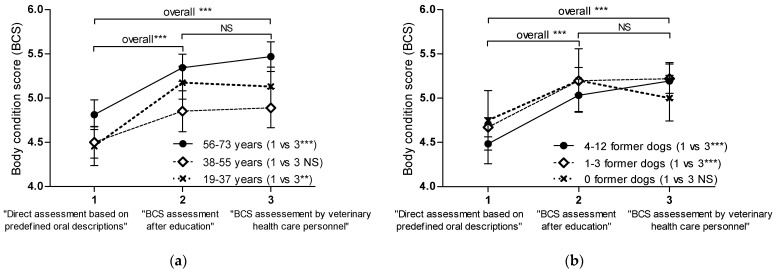
Dog owners’ body condition assessment (time points 1 and 2 on the *x*-axis) and BCS assessment by veterinary health care personnel (time point 3 on the *x*-axis) performed on the same dogs (total numbers of dogs, n = 82) analyzed using a mixed model random analysis. Data are shown as mean ± SEM (**a**,**b**). All groups (except for the group “38–55 years” (n = 27, (**a**) and the group “0 former dogs” (n = 10, (**b**)) significantly underestimated body composition in the direct assessment of their own dogs (time point 1) compared to the trained veterinary health care personnel (time point 3) (** *p* = 0.001 and *** *p* ≤ 0.0003). After the standardized BCS education (time point 2), the overall assessment improves significantly (*** *p* < 0.0001) compared to time point 1, and the BCS assessment is equivalent (NS, *p* ≥ 0.89) to the professional assessment (time point 3) (**a**,**b**).

In the direct assessment of their own dogs based on predefined oral descriptions, the dog owners underestimated their dogs in 29/82 (35%) of the observations, overestimated their dogs in 4/82 (5%) of the observations, and performed equivalent assessment in 49/82 (60%) of the observations. Of the underestimated dogs, 21/29 (72%) were overweight (BCS = 6–7), 6/29 (21%) were of normal weight (BCS = 4–5), and 2/29 (7%) were underweight (BCS = 3). Owner perceptions, in terms of the mean BCS based on predefined oral descriptions and no further education, were significantly lower (4.6 ± 1.0) than the BCS assessed by the veterinary health care personnel (5.2 ± 1.1) in the overall comparison (*p* < 0.0001) (Figure 3). An analysis of the group and time point interactions showed that the direct assessments (with no previous education) of the age group “38–55 years” (Figure 3a) and owners who previously had owned “0 former dogs” (Figure 3b) were equivalent to the professional assessment (*p* = 0.18 and *p* = 0.98, respectively). Owners with no previous experience of dog ownership were few and were almost exclusively from the youngest age group, and all owners aged 38–55 years had previously owned at least one and up to 12 dogs (Table 4).

#### 3.2.3. Owner BCS Assessment after Standardized Education

In terms of the BCS assessment after the standardized education, the dog owners significantly improved in overall BCS assessment (*p* < 0.0001) compared to their previous assessment (Figure 3). The overall (mean ± SD) BCS assessment performed by the dog owners after the education (5.1 ± 1.0) was not different from the mean BCS assessment performed by the veterinary health care personnel (5.2 ± 1.1) (*p* ≥ 0.89) (Figure 3). After the standardized education, the dog owners misclassified their dogs in 19/82 (23%) of the observations and performed equivalent BCS assessments in 63/82 (77%) of the observations. Misclassification were seen primarily for overweight dogs (14/19 dogs), of which ten were underestimated and four were overestimated.

The tabulation of the two explanatory factors, “owner age groups” and “number of previously owned dogs”, is shown in Table 4. Some overlap between the explanatory factors were present, although the number of previously owned dogs differed significantly between age groups (*p* < 0.0001), and increasing age of the owners showed a weak positive correlation (*p* = 0.02, R-squared = 0.06) with increasing number of owned dogs.

#### 3.2.4. Associations of Dog Intrinsic Factors and BCS

Of all participating dogs, 30/82 (37%) were assessed as being overweight (divided into being slightly overweight (BCS = 6, 21/30) and overweight (BCS = 7, 9/30)), 57% was assessed as being of normal weight (BCS = 4–5), and 6% was assessed as being underweight (BCS = 2–3) by the veterinary health care personnel. There was a weak positive association between dog age and BCS, where the BCS of participating dogs increased with increasing dog age (linear regression, *p* = 0.02, R-squared = 0.12). Female dogs had a (mean ± SD) BCS of 5.4 ± 1.0, which did not differ from the BCS of male dogs of 4.9 ±1.1 (*p* = 0.07). Both male and female neutered dogs had a significantly higher (mean ±SD) BCS of 5.7 ± 0.9 compared to intact dogs with a BCS of 5.0 ±1.1 (*p* = 0.006).

## 4. Discussion

This study shows, with consistent results from two different study samples, that owner perceptions of canine body composition seem to be sliding regarding both normal weight and overweight dogs. In the indirect assessment based on photos, the normal-weight dog was underestimated by three-quarters of the owners. In the direct assessment of their own dogs, one-third of the owners underestimated their dogs’ body composition, mainly for dogs with excess adiposity. The results, thus, indicate that an underassessment of normal weight and an inability to identify overweight are two main risks when dog owners evaluate canine body composition without previous education.

### 4.1. Owner Perceptions of Canine Body Composition Evaluated via Indirect Assessment Based on Photos and Direct Assessment of Own Dogs Based on Predefined Oral Descriptions

Assessment of owner perceptions based on photos and in vivo assessment of own dogs were performed in this study with the intention of recording perceptions of canine body compositions without the influence of any standardized system, guiding pictures, or other training. In accordance with several other studies [4,12,21,36,37,38], it is evident that the most common misperception of the dog owners is an underestimation of BCS compared to assessment made by trained veterinary health care personnel. The accuracy of the BCSs assessed using photos was dependent on the BCSs of the dogs, a dog-related factor that has previously been shown to affect dog owner assessments [38,39]. The normal-weight dog was the dog that was most commonly underestimated (74%), followed by the slightly overweight and overweight dogs (51–46%), while the obese dog, on the other hand, was correctly identified by as much as 71% of the survey respondents. Similar results with an underestimation of normal-weight dogs have been previously reported, albeit not based on an assessment of photos but on a direct assessment of own normal-weight dogs [22,23]. The fact that an underestimation of BCS was commonly observed in the survey sample was not surprising [4,12,21,36,37,38], but it was not expected that the underestimation was primarily regarding the dog with an ideal body condition. The challenge of correctly identifying an “ideal body condition” noted in the present study stands in contrast to a similar study investigating owner perceptions via indirect assessment based on photos, which reported an underestimation of BCS mostly with regard to overweight dogs [40].

The obese dog shown in the photo in the present study was of a different color (brown) compared with the other dogs (black). All dogs shown in the photos in the survey were, however, Labrador Retriever dogs of the type for shows, were assessed in vivo by the same primary investigator, and were photographed in a standardized setting. The lighter color could perhaps have contributed to the obese state being more obviously spotted. However, coat color has previously been shown to have no effect on the assessment of BCS when comparing visual assessment using photos to in vivo assessment of the same dogs [40].

The method of visual BCS assessment using photos has been shown to be moderately to highly correlated with in vivo assessment [40,41], but less experienced assessors might vary in their precision [40]. Leptin concentrations in dogs are known to be positively associated with increasing BCS scores [42], and such association has previously been confirmed in the same Labrador Retriever dogs used for indirect BCS assessment of dogs in photos [13], verifying the precision of the in vivo assessment made by the primary investigator. Twelve percent of the survey sample had employment within veterinary health care and were presumably well experienced in BCS assessment; otherwise, the survey sample probably had little previous experience as only 33% could correctly describe what the BCS is. This level of self-perceived knowledge was, however, higher than the results reported in another study showing that only 7% of dog owners had previous knowledge of the BCS scale [21]. Further studies are needed to conclude how difficult indirect assessment based on photos actually is as this method, in comparison to in vivo BCS assessment, excludes one important aspect, palpation. If evaluated as being adequate, assessment using photos could be used for investigating owner perceptions of canine body compositions in larger populations or could be used by veterinary health care personnel in online assessments of client dogs.

### 4.2. Owner-Related Factors Affecting Perceptions of Canine Body Composition

In the indirect assessment using photos, dog owners who had no previous knowledge of the BCS scale and/or were of older age were less likely to perform assessment that was equivalent to the primary investigator, especially in the assessment of the normal-weight dog, where the results differed significantly. In fact, 46 percent of the respondents aged 61+ years in the survey assessed the normal-weight dog (BCS = 5) as being underweight (BCS = 1–3), thus underestimating the dog with an ideal body condition by two steps or more on the scale, which is a clinically significant amount.

In the clinical study sample, overweight dogs were about twice as likely to be underestimated compared to normal-weight/underweight dogs in the pre-educational assessment, a finding that is consistent with other studies investigating the accuracy of BCS assessment among owners evaluating their own dogs [4,21,22,23,37,38]. However, the dog owners of middle age or those who owned a dog for the first time performed the assessment equivalently to the veterinary health care personnel before receiving the standardized education, while the assessment of all other groups differed significantly from that of veterinary health care personnel. The results from the clinical study sample, thus, confirm the finding from the survey sample, showing that the age of owners might be a factor to consider when interpreting the accuracy of owner perceptions regarding different canine body compositions and there is no previous education.

The fact that certain owner-related factors can affect the precision of owner BCS assessments has been confirmed by another study, showing that type of dogs (sport or pet dogs) [37] may affect the precision of assessments. Increasing owner age in the clinical study sample was associated with a slight but significant increase in number of previously owned dogs. Thus, the perception of owners who were more experienced with owning a dog tended to be negatively associated with equivalent body condition assessment, but this result needs further confirmation in larger study populations. A possible explanation for this finding might be that older people with a history of owning multiple dogs could possibly have seen a larger number of overweight or obese dogs and, therefore, tend to underestimate BCS to a larger extent due to unconscious habituation. People owning a dog for the first time were almost exclusively of the youngest age group, and it is likely that this age group might be more updated on the usage of the BCS scale than other participants.

The group of owners with no previous experience of dog ownership was small and did not overlap with the group of owners aged 38–55 years, in which all owners had previously owned at least one dog. The oldest participants in the clinical sample differed in their perceptions compared to the veterinary health care personnel before the standardized education. However, owner age as an explanatory factor for accuracy in assessments could possibly be a marker for some other factors not detectable by the current study design. Importantly, it should be emphasized that all dog owners responded to the standardized education given, regardless of age and previous dog ownership experience. Hence, a recommendation is that veterinary health care personnel should take the time during veterinary visits to provide standardized education to their clients.

Although exact owner ages were grouped slightly differently between the two study samples, the age groups might be considered comparable as they represent similar life stages of the participants. Even though many dog owners participated in this study as a whole, and participants in the clinical study sample were from three different regions of Sweden, the results do not necessarily represent perceptions of Swedish dog owners at a national level due to the use of convenience sampling based on voluntary participation and non-randomized selection. Another limitation of the study is the unintentional overrepresentation of women, health care professionals, and students from animal-related programs in both study samples. The results were not analyzed with regard to owner gender and education, even though one previous study of owner perceptions of canine body composition has shown that women underestimated body composition to a lower degree than men [38], a factor which could have also influenced the results of the current study. Furthermore, a relatively large proportion of the participants worked in veterinary or human health care, which might have contributed to a greater understanding of the BCS scale and its usage. Thus, the results noted here might have been an overall overestimation of people in the general dog owner population (based on randomized selection) who can correctly classify their dogs. Even though quite a large proportion of the dog owners worked in the health care sector, knowledge of some of the comorbidities related to canine overweight, such as reduced life quality and shortened lifespan, was scarce, suggesting that a health care background might not positively affect knowledge and ability regarding factors relevant to dogs’ health.

The results from the two study populations evaluating owner perceptions via indirect assessment based on photos and direct assessment of own dogs based on oral instructions suggested they were influenced by some similar factors, i.e., body condition status of the dogs and owner age. This study shows no overlap between the survey and the clinical samples. In future studies, owner perceptions of what an ideal body condition looks like, as evaluated in dogs owned by others and/or from photos, should be investigated and compared to the accuracy of BCS assessment of a dog owned by the participant in the same study population of dog owners. This will lead to a better understanding of the impact of individual perceptions on the accuracy of BCS assessment.

### 4.3. Canine Overweight Prevalence and Dog Intrinsic Factors

The canine overweight prevalence in the clinical sample was 37%, and of those dogs, about two-thirds were slightly overweight, one-third were overweight, and no dog was obese. A prevalence of nearly 40% is slightly higher than previously reported in Sweden [2,7] but in line with or even slightly lower than canine overweight prevalence reported from the USA [37,43]. However, the sample was based on voluntary participation, and it cannot be ruled out that the included owners had overweight dogs to a higher or lower extent than the average population, i.e., selection bias. However, if slight overweight remains unidentified, it may develop into excess adiposity, which is much more challenging to treat [44] and with more severe health consequences for dogs. Therefore, even slightly overweight as observed in the clinical sample should receive attention. Nevertheless, as the overweight prevalence is reported to have increased [33], the risk of perceiving excess adiposity as an ideal body condition may increase accordingly. The best prevention and treatment measure is to ensure that dog owners can accurately identify when the body condition of their dogs starts to increase beyond a normal weight.

In this study, canine BCS increased with the age of the dogs and neutered dogs had a significantly higher BCS than intact dogs. The fact that these intrinsic dog factors affect BCS has previously been verified in other studies [3,30,45,46,47], and should be continuously communicated to dog owners as risk factors at veterinary consultations. Owner-related factors affecting canine overweight development, such as owner overweight and knowledge of the obesity problem, show that canine overweight is not a problem solely for dogs, but rather a one health issue that is delicately dependent on the human–animal bond and a shared lifestyle [2,27,28,30,31,48,49], where owners’ views on diet and exercise can affect their dog which cannot take on responsibility for its own body condition. The ability of dog owners to adequately assess body condition and identify incipient adiposity in their dogs is, therefore, a vital key factor for increased awareness of canine overweight and is necessary in order to perform adjustments in energy intake and/or physical activity level to prevent overweight and reach or maintain a normal weight [20].

### 4.4. Effect of Standardized Education on Owner BCS Assessment

Both study samples underestimated body condition at assessment when they had no previous education; however, after the standardized practical BCS education given to the dog owners in the clinical sample, these owners performed the BCS assessments equivalent to the veterinary health care personnel overall, regardless of owner age and/or number of previously owned dogs. The standardization of the education is unique to the authors’ knowledge and likely contributed to the significantly improved agreement between the assessments from the dog owners and the veterinary health care personnel. This suggests that a systematic education could be vital for owners’ ability to correctly assess their dogs’ body condition.

Other studies have reported no or only little improvement in the accuracy of BCS assessment after using the BCS scale and/or after some training directed to dog owners [4,21,22,23], which stands in contrast to the results of the current study. In one of these previous studies, dogs were more accurately assessed only with regard to overweight dogs (BCS = 4/5) [22], whereas in another other study [21], improved assessment could only be detected for underweight dogs (BCS = 1–2/5). In these two studies, owners were given the BCS scale on paper with no further education and the 5-point BCS scale was used, which might reduce precision in the assessments since each level on the scale covers a larger variety of body compositions. In the clinical study sample of the current study, the owners were given a standardized education on how to use the 9-point BCS scale (oral and written information) including a practical anatomic demonstration, and the results showed that accuracy improved significantly from 60 to 77%. In two other studies evaluating owner assessments using the 9-point BCS scale, with additional oral explanation, about 30–40% of normal-weight dogs (BCS = 4–5) and slightly overweight to overweight dogs (BCS = 6–7) were underestimated [4,23]. This is comparable to the numbers observed in the clinical study sample in this study before any education was received. After the education, the present study found a misclassification of only 23%.

We suggest that the concept of “weight blindness”, defined here as an underassessment of normal-weight dogs and an inability to identify overweight dogs, could be introduced as a novel concept. Weight blindness captures the bias underlying the underestimation of canine body condition and is likely to have a negative impact on canine overweight prevalence as owners will not address an unknown problem. In accordance with other studies [4,41], we suggest that deeper knowledge about owner perceptions of different canine body compositions can inform the development of new strategies to help prevent and manage canine overweight; whereof the standardized practical education on BCS assessment shown here is one good example.

## 5. Conclusions

The results from the two different study samples indicate that owner perceptions of an “ideal body condition” in dogs are sliding and that the ability to identify overweight in one’s own dogs might be limited. We propose a concept called “weight blindness”, which could be successfully reversed with standardized practical education on how to use the 9-point BCS scale. When addressing canine overweight, it should be recognized that not only the body condition of dogs but also owner-related factors might influence owners’ perceptions of different canine body compositions and the accuracy of BCS assessment when there is no previous education. Future studies should preferably investigate perceptions among owner populations based on a randomized selection of participants. Dogs owned by others and/or perceptions based on photos, as well as assessments of one’s own dogs, in the same study population of owners could be used to generate a better understanding of the impact of individual perceptions on the accuracy of BCS assessment.

## Figures and Tables

**Table 1:** Detailed descriptive data of the survey sample, n^dog owners^ = 564.

Owner Data	Number	%
Gender		
Male	17	3
Female	543	96
Non-binary	4	1
Age		
18–25 years	52	9
26–40 years	207	37
41–60 years	253	45
≥61 years	70	12
Educational level		
Upper secondary school	296	52
University 1–3 years	127	23
University > 3 years	132	23
PhD or higher	9	2
Employment		
Health care	121	21
Veterinary health care	67	12
Teaching	44	8
Sales	34	6
Economy	31	6
Administration	30	5
Social work	28	5
IT	21	4
Categories with <20 participants *	91	16
Other (unspecified)	97	17
Experience of owning dogs		
First-time dog owner	64	11
1–3 previous dogs	192	34
4–12 previous dogs	242	43
≥13 previous dogs	66	12
Previous self-perceived knowledge of BCS		
Yes (with correct description in free-text answer)	188	33
Yes (with incorrect description in free-text answer)	36	7
No	340	60

* The categories are industrial sector (n = 18), agricultural sector (n = 14), culture/media/design (n = 11), transportation and logistics (n = 11), restaurant and hotel services (n = 11), sanitation and cleaning (n = 7), construction (n = 6), law (n = 6), craft professions (n = 3), installation/management/maintenance (n = 2), beauty sector (n = 1), and environmental sector (n = 1).

**Table 2:** Cross tabulation of the explanatory factors, “owner age groups” and “previous knowledge of a BCS scale”, in the survey sample. The proportions (Familiar: yes/no) do not differ significantly between age groups (Chi-square test for trend, *p* = 0.09).

Age Group of Owners	Previous Knowledge of the BCS Scale	
	Familiar: Yes	Familiar: No
18–25 years	25	27
26–40 years	83	124
41–60 years	97	138
≥61 years	19	51

**Table 3:** Detailed descriptive data of the clinical sample, n^dog owners^ = 64 and n^dogs^ = 82.

Owner Data	Number	%
Gender		
Female	49	76
Male	14	22
Non-binary	1	2
Age		
19–37 years	19	30
38–55 years	20	31
56–73 years	25	39
Employment		
Retired	14	22
Health care	8	13
Student (animal programs)	7	11
Pedagogical work	6	9
Student (other programs)	5	8
Unemployed	2	3
Other dog-related work	2	3
Other (unspecified)	20	31
**Dog Data**	**Number**	**%**
Gender		
Intact female	33	40
Neutered female	13	16
Intact male	22	27
Neutered male	14	17
Age		
<1 year	11	13
1–3 years	27	33
4–8 years	28	34
≥9 years	16	20

**Table 4:** Cross tabulation of the explanatory factors, “owner age groups” and “number of previously owned dogs”, in the clinical sample. Number of previously owned dogs differs significantly between age groups (Chi-square test for trend, *p* < 0.0001), and the age of the owners and number of owned dogs show a weak positive correlation (linear regression, *p* = 0.02, R-squared = 0.06).

Age Group of Owners	Number of Previously Owned Dogs		
	No Former Dogs	1–3 Former Dogs	4–12 Former Dogs
19–37 years	8	8	7
38–55 years	0	21	6
56–73 years	2	12	18

## Data Availability

The data presented in this study are available in the article or in the Supplementary Materials.

## Supplementary Materials

The following supporting information can be downloaded at https://www.mdpi.com/article/10.3390/vetsci10070447/s1. Table S1: Survey questions and research raw data; Supplementary Material S1: Standardized information; Figure S1: Dog photos.
