# Supplementary material for: Dog Owners’ Perceptions of Canine Body Composition and Effect of Standardized Education for Dog Owners on Body Condition Assessment of Their Own Dogs
Source: Vet Sci. 2023 Jul 8;10(7):447. doi: 10.3390/vetsci10070447 (PMC10386090; doi:10.3390/vetsci10070447)
Supplement: Supplementary file 1 [file vetsci-10-00447-s001.zip › Figure S1. Dog photos.pdf]

Body condition score (BCS)

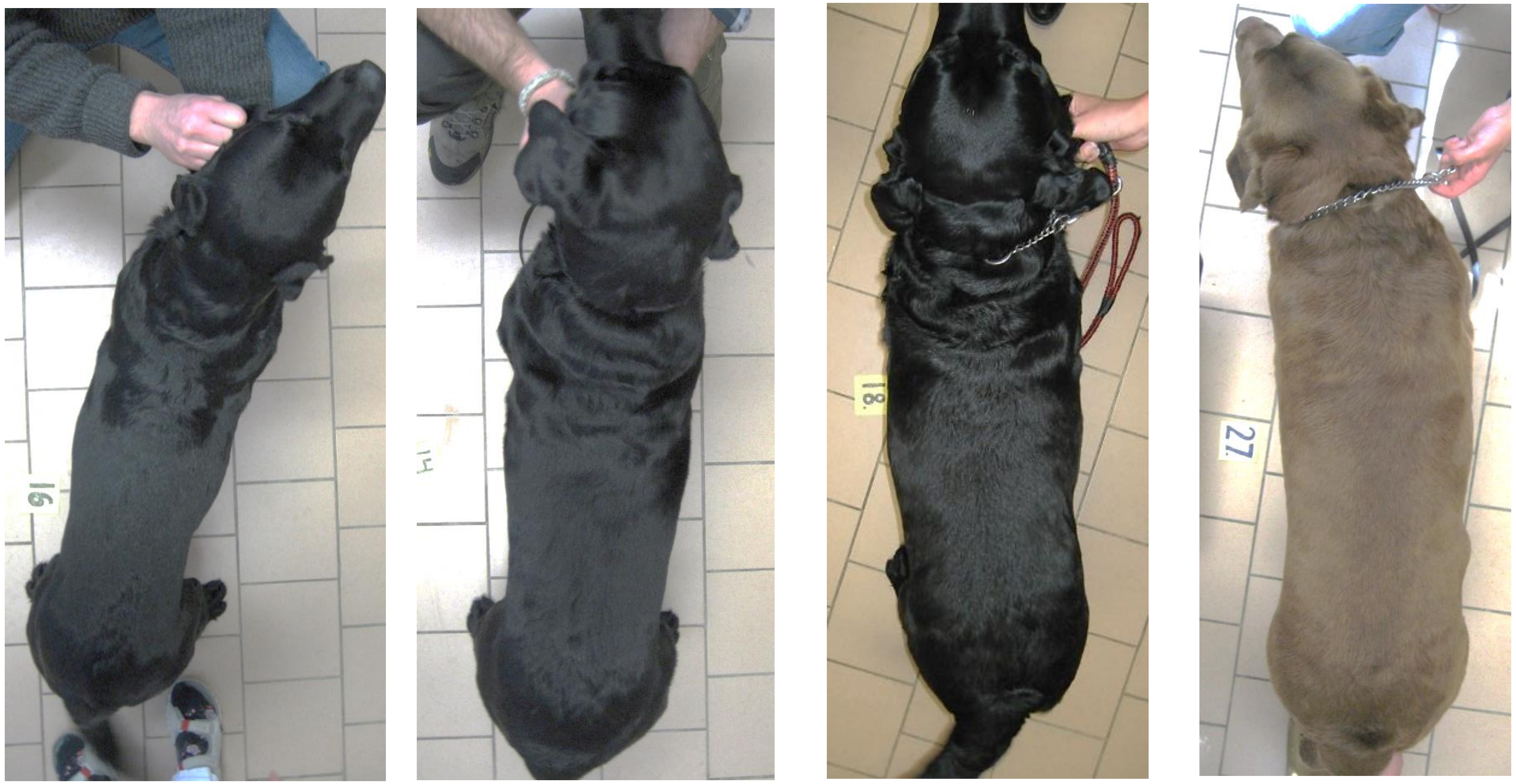

BCS 5 BCS 6 BCS 7 BCS 8

Figure S1. Photos of Labrador retriever dogs displayed in the online survey, here showed in increasing body condition score (BCS), from left to right. Photos originally published in thesis by Söder, J. Doctoral thesis. *Metabolic variations in canine overweight.* Acta Universitatis Agriculturae Sueciae. Swedish University of Agricultural Sciences, Department of Anatomy, Physiology and Biochemistry, 2018. Photos: Josefin Söder.
