# Supplementary material for: Dog Owners’ Perceptions of Canine Body Composition and Effect of Standardized Education for Dog Owners on Body Condition Assessment of Their Own Dogs
Source: Vet Sci. 2023 Jul 8;10(7):447. doi: 10.3390/vetsci10070447 (PMC10386090; doi:10.3390/vetsci10070447)
Supplement: Supplementary file 1 [file vetsci-10-00447-s001.zip › Supplementary Material S1. Standardized information.pdf]

Table S2. Muntlig och skriftlig information

Framför dig ser du en hullbedömningsskala som är graderad från 1–9 där ett motsvarar kraftig undervikt och nio motsvarar fetma. 1–3 representerar olika grader av undervikt, 4–5 representerar normalvikt och 6–9 motsvarar olika grader av övervikt. Observera att du endast kan ge hela poäng. Alla olika poäng mellan 1–9 kan tilldelas, även de som saknar bild.

Du ska titta på hunden ovanifrån och från sidan samt känna på hunden med händerna. Du jämför din hund med hullbedömningsskalans text och bild och kommer fram till en poäng för din hund.

Den poäng du väljer att tilldela hunden ska grundas på både det du ser (din visuella bedömning) OCH det du känner med händerna

När du ska känna på din hund ska det alltid vara med ett lätt tryck och hela handflatan (visa handflatan).

Fokus under hullbedömningen ligger på hur tydligt revbenen känns och hur tydligt midjan och buklinjen framträder (Visar på hunden de tre "områdena", revben, midja och buklinje). Din tilldelade poäng för din hunds hull blir en sammanvägd poäng för dessa tre områden.

För bröstkorgen: Är revbenen synliga eller icke synliga? Är revbenen lätta eller svåra att känna med dina händer, eller går de inte att känna alls? Jämför din hund med hullbedömningsskalans text och bild.

För midjan: Titta på midjan ovanifrån. Är midjan tydlig, saknas midjan (hunden är rak) eller är buken utspänd? Jämför din hund med hullbedömningsskalans text och bild.

För buklinjen: Titta från sidan. Är buklinjen tydligt uppdragen, uppdragen eller inte uppdragen alls? Jämför din hund med hullbedömningsskalans text och bild.

När du är färdig med din hullbedömning noterar du hundens poäng i formuläret och lämnar över det till försöksledaren. Därefter gör försöksledaren sin hullbedömning av din hund.
